# Targeting aldehyde dehydrogenase activity in head and neck squamous cell carcinoma with a novel small molecule inhibitor

**DOI:** 10.18632/oncotarget.17017

**Published:** 2017-04-10

**Authors:** Jeewon Kim, June Ho Shin, Che-Hong Chen, Leslie Cruz, Lovisa Farnebo, Jieying Yang, Paula Borges, Gugene Kang, Daria Mochly-Rosen, John B. Sunwoo

**Affiliations:** ^1^ Stanford Cancer Institute, School of Medicine, Stanford University, Stanford, CA, 94305, USA; ^2^ Division of Head and Neck Surgery, Department of Otolaryngology, Stanford University, Stanford, CA, 94305, USA; ^3^ Department of Chemical and Systems Biology, Stanford University, School of Medicine, Stanford, CA, 94305, USA; ^4^ Division of Otorhinolaryngology, Department of Clinical and Experimental Medicine, Faculty of Health Sciences, Linköping University, SE-58185, Linköping, Sweden; ^5^ Department of Developmental Biology, Stanford Institute for Stem Cell Biology and Regenerative Medicine, Stanford University School of Medicine, Stanford, CA, 94305, USA

**Keywords:** aldehyde dehydrogenase, chemoresistance, head and neck squamous cell carcinoma, small molecule inhibitors

## Abstract

Chemoresistant cancer cells express high levels of aldehyde dehydrogenases (ALDHs), particularly in head and neck squamous cell carcinoma (HNSCC). The ALDH family of enzymes detoxify both exogenous and endogenous aldehydes. Since many chemotherapeutic agents, such as cisplatin, result in the generation of cytotoxic aldehydes and oxidative stress, we hypothesized that cells expressing high levels of ALDH may be more chemoresistant due to their increased detoxifying capacity and that inhibitors of ALDHs may sensitize them to these drugs. Here, we show that overall ALDH activity is increased with cisplatin treatment of HNSCC and that ALDH3A1 protein expression is particularly enriched in cells treated with cisplatin. Activation of ALDH3A1 by a small molecule activator (Alda-89) increased survival of HNSCC cells treated with cisplatin. Conversely, treatment with a novel small molecule ALDH inhibitor (Aldi-6) resulted in a marked decrease in cell viability, and the combination of Aldi-6 and cisplatin resulted in a more pronounced reduction of cell viability and a greater reduction in tumor burden *in vivo* than what was observed with cisplatin alone. These data indicate that ALDH3A1 contributes to cisplatin resistance in HNSCC and that the targeting of ALDH, specifically, ALDH3A1, appears to be a promising strategy in this disease.

## INTRODUCTION

About 650,000 new cases of HNSCC arise each year worldwide, and the 5-year survival rate for advanced non-human papilloma virus (HPV)-associated HNSCC has remained at approximately 50% for the last 20 years [1–3]. Only approximately 30% of patients are diagnosed at an early stage, and most patients present with advanced disease and lymph node metastasis [1–3]. Current standard of care involves multiple modalities of treatment, including surgery, chemotherapy, and radiation. Cisplatin is currently the most commonly used chemotherapeutic agent for HNSCC [1]. However, cisplatin resistance and a significant incidence of toxic side effects (*e*.*g*., ototoxicity and nephrotoxicity) pose serious issues in the management of this disease [1, 2, 4–6].

Several signaling pathways involved in the development of cisplatin resistance in cancer have been identified. These include increased inactivation by cellular antioxidants and antioxidant enzymes (Glutathione (GSH) and Glutathione S-Transferase (GST)); increased cisplatin efflux by upregulated P-type ATPases; overexpression of multidrug resistance protein (MRP2), a member of ABC membrane transporters that mediate the ATP-dependent cellular efflux of cisplatin; reduced uptake by downregulated plasma membrane copper transporter (CTR1); decreased efficacy by increased expression of cisplatin binding proteins (e.g., voltage dependent anion channel (VDAC)); and upregulation of antiapoptotic members of the Bcl2 proteins [7–10]. Due to the heterogeneous nature of resistance to cisplatin, effective ways to sensitize HNSCC to cisplatin have not been developed [9].

Aldehyde dehydrogenase (ALDH) is a superfamily of 19 human isoforms that metabolizes reactive aldehydes produced from alcohol, chemotherapeutic compounds and lipid peroxidation, into non-reactive acids [11–13]. Lipid peroxidation refers to the oxidative degradation of lipid membranes, which generates hundreds of types of reactive aldehydes, including 4-hydroxy 2-nonenal, malondialdehyde and acrolein methylglyoxal, many of which are highly cytotoxic [14]. In both cancer and neurodegenerative diseases, increased level of oxidized macromolecules from reactive oxygen species (ROS) has been well documented [11, 15]. Many chemotherapeutic drugs, including cisplatin and erlotinib, are also known to generate oxidative stress and elevate levels of lipid peroxidation derived aldehydes [16, 17]. ALDHs play a critical role in metabolizing these reactive aldehydes and reducing oxidative stress in the cells [18].

The fact that mutations in various ALDH genes and altered expression of these genes are implicated in multiple cancers highlights the importance of the breakdown of oxidizing aldehydes to non-toxic products as a critical process to reduce oxidative stress. In many types of cancer, especially in HNSCC [19–21], ALDH activity is elevated in subpopulations of cells that are chemo/radiotherapy-resistant, such as putative cancer stem cells (CSC). Furthermore, high levels of ALDH1 in patient samples have been correlated with poor prognosis in HNSCC [21], lung [22], prostate [23], breast [24] and pancreatic cancer [25]. Increased ALDH1A1 and ALDH3A1 activity is a predictor of decreased efficacy of cyclophosphamide treatment in breast adenocarcinoma [26, 27]. Cyclophosphamide or mafosfamide was shown to be metabolized to aldophosphamide in the target cells and was found to be further metabolized to the toxic compounds phosphoramide mustard and acrolein or to a nontoxic metabolite carboxyphosphamide by ALDH3A1 isozymes [28]. These data suggest that increased ALDH levels play an important role in chemoresistance through the detoxification of the chemotherapeutic compound metabolites. Thus, isozyme-selective inhibitors of the relevant ALDHs may reduce resistance to the chemotherapy in cancer cells and sensitize these cells to lower doses of chemotherapeutic agents.

Effective isozyme-specific inhibitors of ALDH, however, have not been available until recently [13, 29]. We have previously shown that small molecule ALDH inhibitors (“Aldis” for al*dehyde* d*ehydrogenase* i*nhibitor*s) can increase the sensitivity of the lung cancer cell line A549 to the cytotoxic effects of mafosfamide (a metabolite of cyclophosphamide), possibly by inhibiting the metabolism of the chemotherapeutic drug into its inactive metabolite [30].

Here, we sought to determine if Aldi can sensitize HNSCC to cisplatin treatment. Our data demonstrate that ALDH3A1 increases cisplatin resistance in HNSCC and suggest that targeting this enzyme with a novel isozyme-specific inhibitor is a potentially viable treatment strategy.

## RESULTS

### Cisplatin increases ALDH activity and ALDH3A1 expression in HNSCC

**Figure 1:**
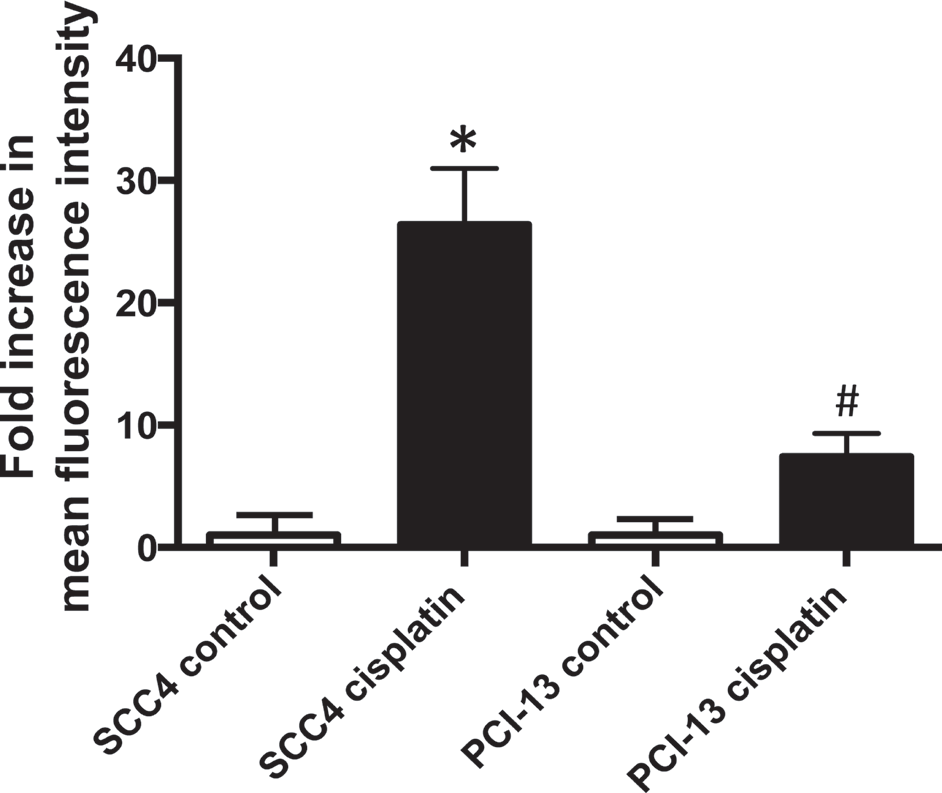
Cisplatin increases ALDH activity in HNSCC SCC4 and PCI-13 cells were treated for 2 days with 15 μM cisplatin. ALDH activity was determined using an Aldefluor assay in surviving cells and compared with the non-treated cells to evaluate the effect of cisplatin. The fluorescence intensity (ALDH activity) was determined by flow cytometry. Results represent the means and SEMs of two independent experiments. (* and ^#^*p* < 0.05 compared to respective controls, *t*-test).

ALDH activity in human HNSCC cell lines was assessed using an Aldefluor-based flow cytometric assay. Although low ALDH activity was measured in the cell lines at baseline, exposure of the cells to cisplatin resulted in a significant increase (Figure 1). When the oral cavity squamous cell carcinoma derived cell line, SCC4, was treated with cisplatin in culture, we observed a 26-fold increase in ALDH activity in the treated cells compared to control cells. Similarly, when another oral cavity squamous cell carcinoma cell line PCI-13 was treated with cisplatin in culture, we observed a 7-fold increase in ALDH activity, in treated cells compared to control. Among the HNSCC cells we screened, SCC4 and PCI-13 cells showed moderate to strong degree of resistance to cisplatin in a cell survival MTT assay (Supplementary Figure 1A and 1B), whereas SCC6 and SCC103 showed stronger resistance to cisplatin based on their IC_50_ (Supplementary Figure 1C and 1D). We first selected SCC4 and PCI-13 to see if the cisplatin resistance in these cells can be improved with Aldis.

**Figure 2:**
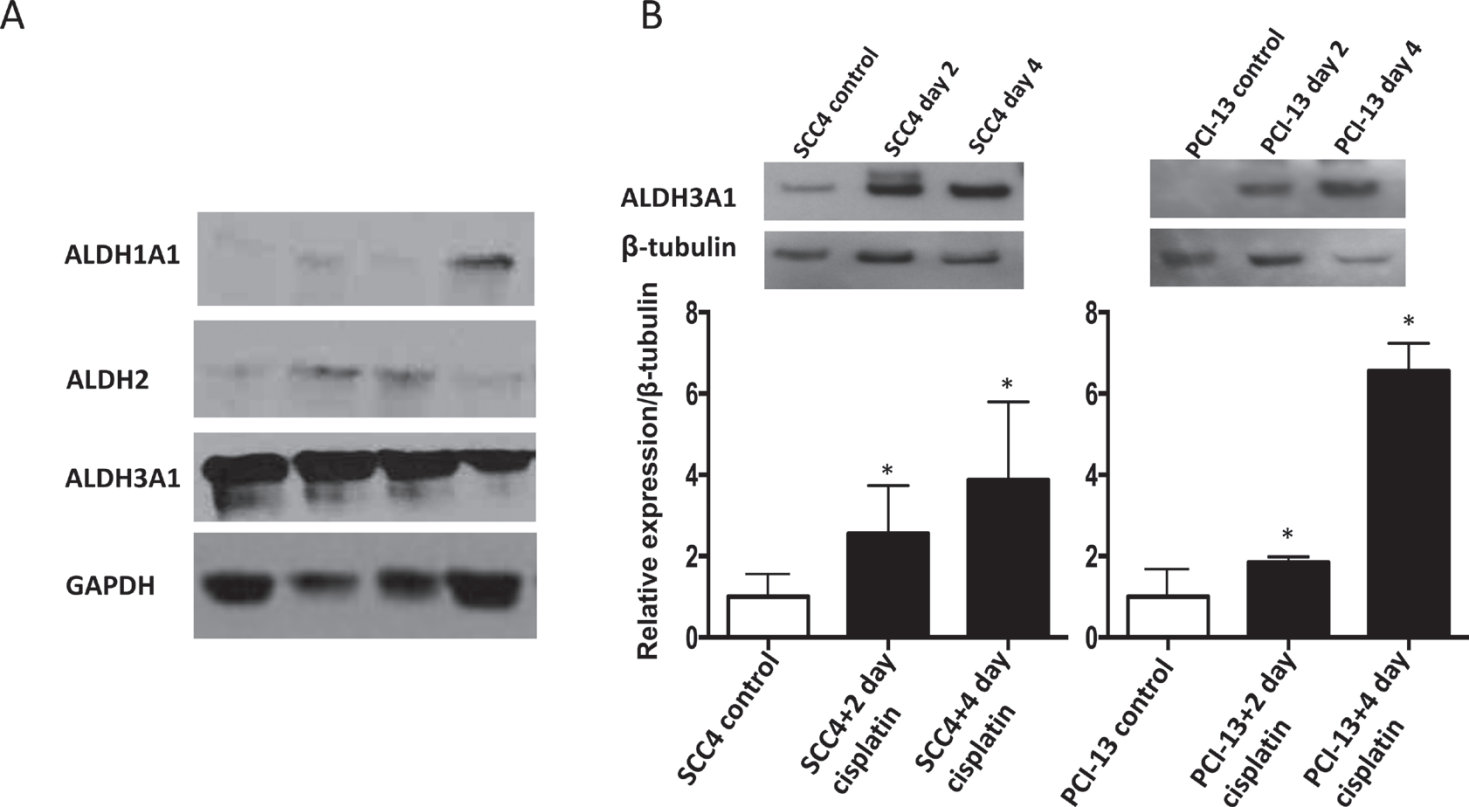
Cisplatin increases ALDH3A1 expression in HNSCC (**A**) Western blot analyses of human primary tumor homogenates from HNSCC patients using GAPDH as a loading control. (**B**) SCC4 and PCI-13 cells were treated with cisplatin (15 μM) for 2 and 4 days and total cell lysates were analyzed by Western blot for ALDH3A1 protein. Densitometric analysis of ALDH bands obtained by Western blot using Image J software shows relative levels after normalization for equal protein loading using β-tubulin as a loading control. Results are expressed as mean±SEM. (*p < 0.05 *vs*. respective control, *t*-test).

To investigate the specific isoform of ALDH that may be responsible for the increased ALDH activity observed in the cisplatin-treated cells, we examined ALDH1A1, ALDH2 and ALDH3A1 expression at the protein level in primary oral cavity squamous cell carcinoma samples from patients. We focused on ALDH1A1, ALDH2 and ALDH3A1 expression among the various isoforms because ALDH1A1 and ALDH3A1 have been shown to be markers of resistance to cyclophosphamide in breast and lung cancer cells [26, 27, 30] and because ALDH2 is a major detoxifying enzyme for reactive aldehydes [15]. Of these isoforms, ALDH3A1 was found to be the predominant isoform that was expressed in the primary tumor samples, and this expression was at a much higher level than that of ALDH1A1 or ALDH2 (Figure 2A). Furthermore, cisplatin treatment of the SCC4 and PCI-13 cells resulted in a marked increase in ALDH3A1 protein expression (Figure 2B) and in transcription in SCC4 cells (Supplementary Figure 2), indicating that this isoform may be responsible for the increased ALDH activity observed in these cells after cisplatin treatment (Figure 1). Importantly, we did not observe a similar increase in ALDH1A1 or ALDH2 protein levels after cisplatin treatment (Supplementary Figure 3A and 3B). While it is possible that ALDH3A1 protein expression was induced by cisplatin, we also noted that cisplatin, at the concentration (15 μM) used here, was sufficient to result in 40% and 60% cell death in SCC4 and PCI-13, respectively (Supplementary Figure 1A and 1B). Thus, it is highly likely that the more cisplatin-resistant cells had higher ALDH3A1 expression and activity.

### ALDH3A1 activation enhances cisplatin-resistance in HNSCC

**Figure 3:**
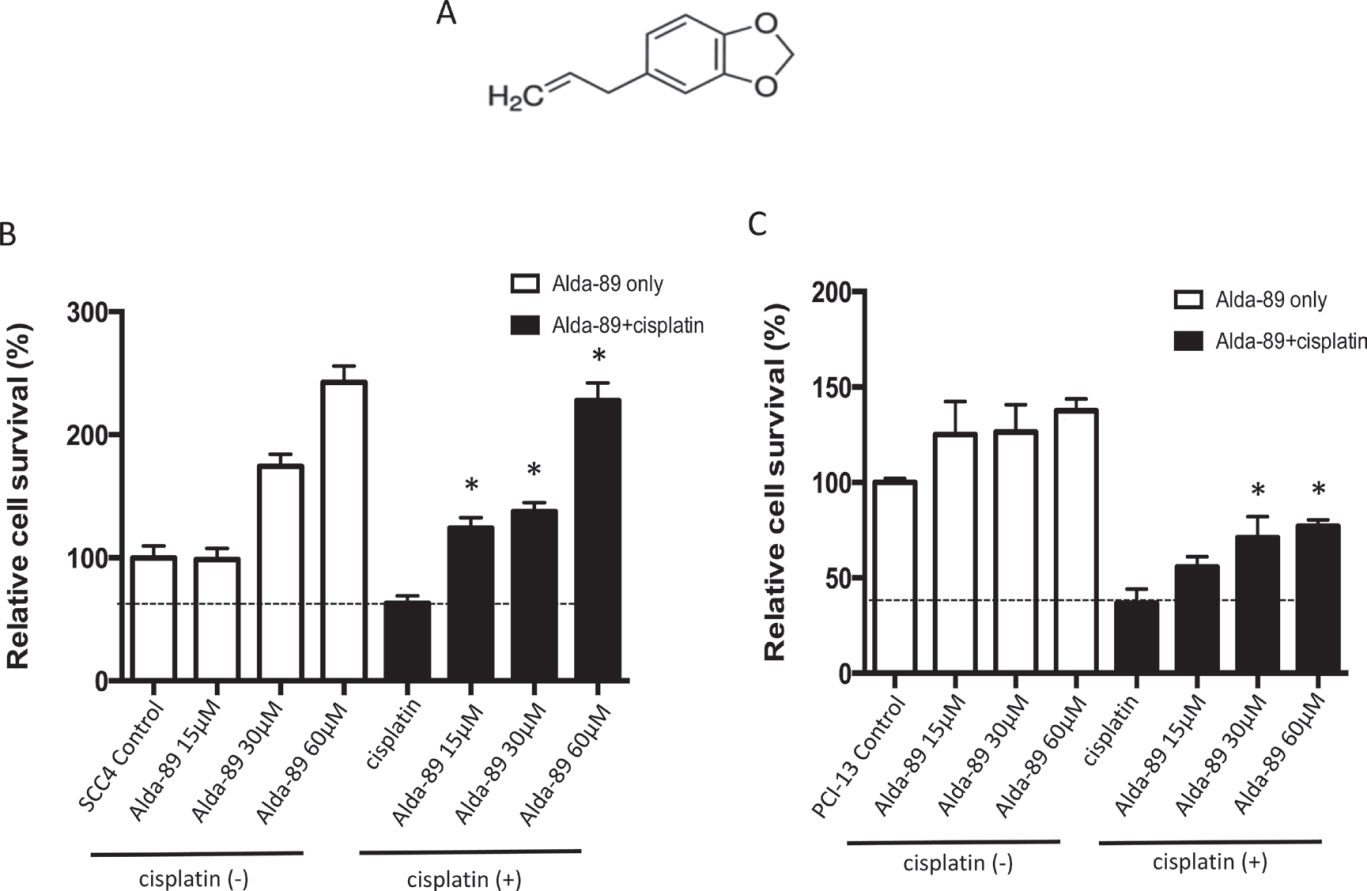
ALDH3A1 activation enhances cisplatin-resistance in HNSCC (**A**) Structure of Alda-89, a small molecule ALDH3A1 activator, is shown (4-Allyl-1,2-methylenedioxybenzene, MW 162.1). (**B**–**C**) SCC4 cells and PCI-13 cells were treated with increasing concentrations of Alda-89 (15–60 μM) and/or cisplatin (15 μM) for four consecutive days. Then, the cells were analyzed on the fourth day. The percentage of live cells is shown compared to that of control cells. Cell viability was quantified using MTT assay, which was performed in 4–8 replicates in two independent experiments. Results represent mean ± SEMs (*p < 0.05 *vs*. respective cisplatin-only controls (*t*-test)).

To investigate the role of ALDH3A1 in conferring cisplatin resistance, we utilized a novel activator of ALDH3A1, called “Alda-89” (4-Allyl-1,2-methylenedioxybenzene, MW 162.1, Figure 3A), which our group has previously reported [4, 31]. In those studies, the specific activity of Alda-89 was extensively tested on ALDH isozymes, including ALDH1A1, ALDH2*1, ALDH2*2, ALDH3A1, ALDH3A2, ALDH4A1, ALDH5A1 and ALDH7A1 [31]. Alda-89 was found to be an activator for ALDH3A1 among the eight ALDH isozymes tested. Importantly, among the 19 ALDH isozymes, ALDH3A2, which arises from a gene duplication event in tandem and shares the highest homology to ALDH3A1 (68% amino acid identity), did not respond to Alda-89 [31]. It is, therefore, more than likely that Alda-89 would not have an effect on other more divergent ALDH isozymes.

When Alda-89 was incubated alone with SCC4 cells, a dose-dependent increase in the cell viability was observed (Figure 3B, left, white bars). Treatment of SCC4 cells with Alda-89 in combination with cisplatin for four consecutive days resulted in a dose-dependent increase in cell survival (Figure 3B, right, black bars), indicating that activation of ALDH3A1 can increase cisplatin resistance in SCC4 cells (Figure 3B). Similar results were observed with PCI-13 cells (Figure 3C). Thus, the ALDH3A1 isoform, which is enriched when HNSCC cells are exposed to cisplatin (Figure 2), appears to contribute to cisplatin resistance.

### Aldi-6 inhibits ALDH activity in HNSCC

**Figure 4:**
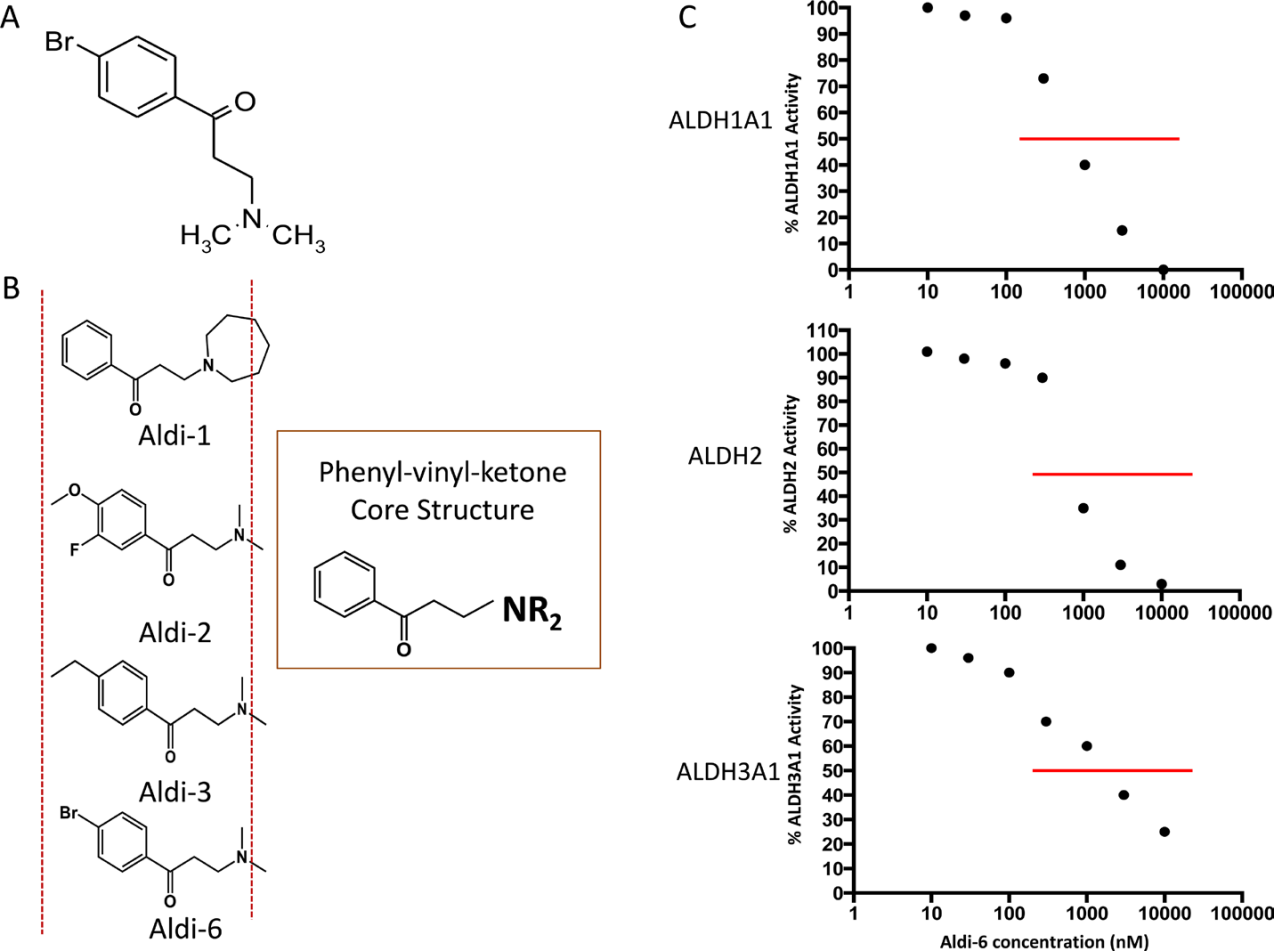
Aldi-6 and inhibitory activity against ALDH (**A**) Aldi-6 structure is shown (3-(Dimethylamino)-4′-bromopropiophenone, MW 261). (**B**) Common core structure of phenyl-vinyl-ketone in Aldis-1, 2, 3 and 6 is shown. (**C**) IC_50_ values of ALDH were determined by an activity assay with purified ALDH1A1, 2 and 3A1 isozymes with Aldi-6 (1–100,000 nM).

To investigate if the inhibition of ALDH3A1 can enhance cisplatin toxicity, we used a novel ALDH inhibitor (3-(Dimethylamino)-4′ bromopropiophenone, MW = 261), discovered by our group and herein, referred to as Aldi-6 (Figure 4A). It has the same core structure as Aldi-1, 2 and 3, which our group has previously described (Figure 4B) [30], and thus, Aldi-6 is believed to utilize a similar molecular mechanism in inhibiting ALDH. Aldi-6 inhibits ALDH3A1, as well as ALDH1A1 and 2, with an IC_50_ of 600 nM for ALDH1A1, 800 nM for ALDH2, 1,000 nM for ALDH3A1 (Figure 4C).

**Figure 5:**
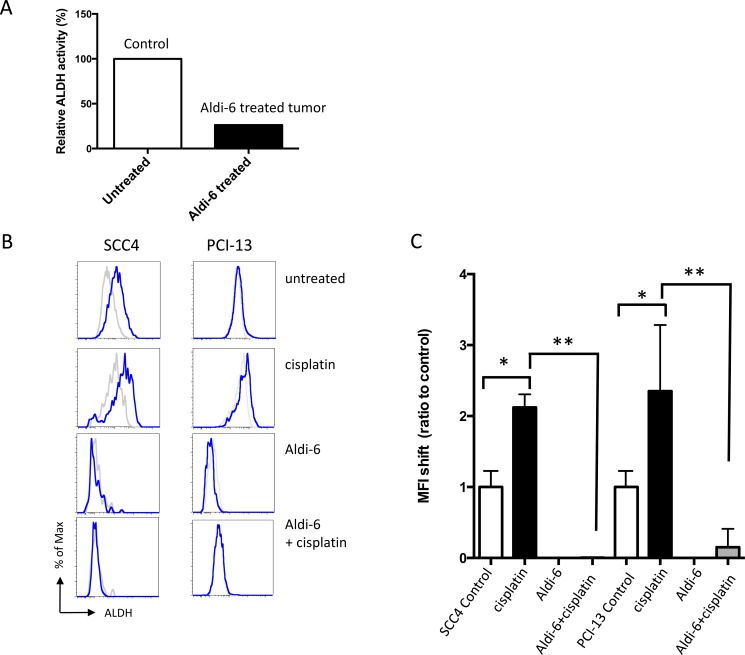
Aldi-6 inhibits ALDH activity in HNSCC (**A**) ALDH3A1 activity in SCC4 xenograft tumor. Mice with SCC4 xenografts were treated intra-tumorally with Aldi-6 or vehicle control for three consecutive days, and the ALDH3A1 activity was measured using an isoelectric focusing assay (see Methods). Experiment was performed three times. (**B**) Representative FACS analyses of ALDH activity of SCC4 and PCI-13 cells. After a two-day treatment of cisplatin (0.88 μM) and/or Aldi-6 (30 μM), ALDH activity was measured in the surviving cells by Aldefluor assay. Grey line represents the DEAB-treated negative control for each treatment condition. Blue line represents the ALDH activity of each sample. (**C**) Changes in ALDH activity in (B) were quantified as a ratio of the shift of MFI between treated and untreated sample. The ratio in MFI shift was calculated by (MFI of treated sample-(MFI of treated sample+DEAB))/(MFI of untreated sample-(MFI of untreated sample+DEAB)). Results represent the means ± SEMs of 2–3 independent experiments with 10,000 cells each. (*p < 0.05 *vs*. untreated control and **p < 0.05 *vs*. cisplatin control (*t*-test)).

We assessed the ability of Aldi-6 to inhibit ALDH3A1 activity in SCC4 HNSCC xenografts. Mice with established SCC4 xenografts were treated intra-tumorally with Aldi-6 daily for 3 days (40 μg/tumor/day). The ALDH3A1 activity was measured using an in-gel isoelectric focusing method, as previously described [31], that separates ALDH proteins by their isoelectric points and measures the amount of NADH produced which is representative of the enzyme activity. We observed a 70% reduction in the ALDH3A1 activity in the treated tumor lysate (Figure 5A). To further investigate the efficacy of Aldi-6 on ALDH3A1, we also treated SCC4 cells with Aldi-6, cisplatin, or both and assessed ALDH activity by Aldefluor assay (Figure 5B and 5C). The choice of the Aldi-6 concentration was based on the efficacy of this compound on SCC4 and PCI-13 cells. In the range of concentrations used, there was 20% reduction in cell survival in SCC4 and about 60% reduction in PCI-13 cells at 30 μM of Aldi-6 (Supplementary Figure 4). With cisplatin treatment, ALDH activity increased by about two-fold in both SCC4 and PCI-13 cells, as measured by mean fluorescence intensity (MFI) (Figure 5C). With Aldi-6 treatment alone (30 μM), ALDH activity became undetectable. Furthermore, in cisplatin-treated cells, Aldi-6 profoundly reduced the cisplatin-induced ALDH activity (Figure 5C and Supplementary Figure 5). Although our ALDH activity assays are not able to discriminate the activities of the different ALDH isoforms, considering our previous observations that ALDH3A1 is the principal constitutive and cisplatin-induced isoform (Figure 2 and Supplementary Figure 3), the data indicate that Aldi-6 may be a highly potent inhibitor of this isoform.

### Inhibition of ALDH3A1 sensitizes HNSCC cells to cisplatin

**Figure 6:**
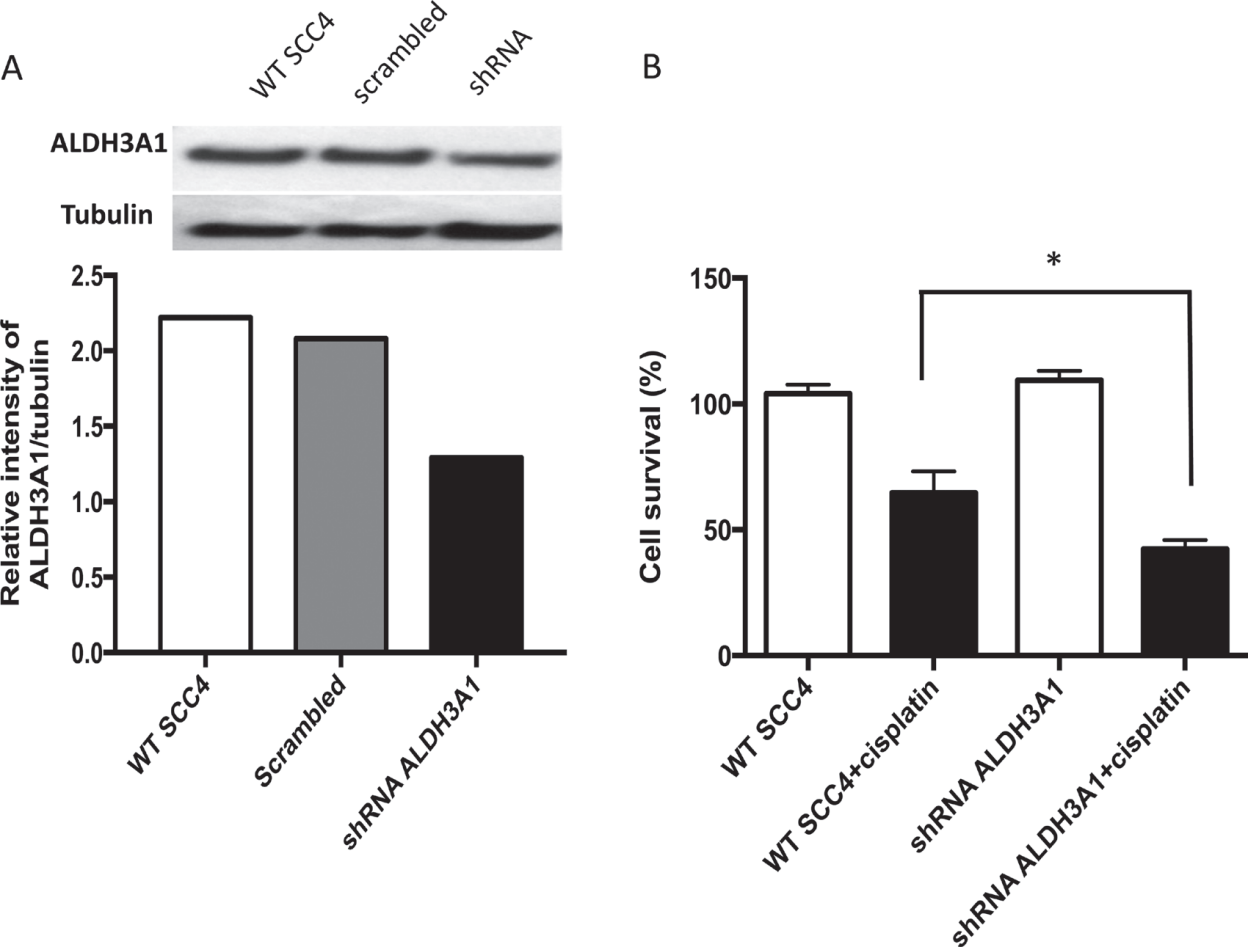
Knockdown of ALDH3A1 expression in HNSCC cells increases sensitivity to cisplatin (**A**) Knockdown of ALDH3A1 by lentiviral transduction of shRNA in SCC4 cells was confirmed by Western blot assay. (**B**) Wild type (WT) and ALDH3A1 knockdown cells were treated with cisplatin on days 1 and 2, and cell viability was quantified by MTT on the fourth day. Results were expressed as percent of control (*p < 0.05 *vs*. cisplatin treated control, *t*-test).

To investigate the role of ALDH3A1 in cisplatin resistance, we used lentiviral transduction of shRNA to knock down expression of this isoform. The amount of ALDH3A1 protein was assessed in SCC4 cells transduced with either shRNA targeting ALDH3A1 or scrambled control shRNA using Western blot (Figure 6A). The level of ALDH3A1 protein decreased by about 40%. We then assessed the cell viability of transduced SCC4 cells without and with cisplatin. Transduction of ALDH3A1 shRNA significantly enhanced cisplatin sensitivity and led to a reduction in cell viability (Figure 6B). Together, these knockdown data indicate that ALDH3A1 plays an important role in the resistance of SCC4 cells to cisplatin.

**Figure 7:**
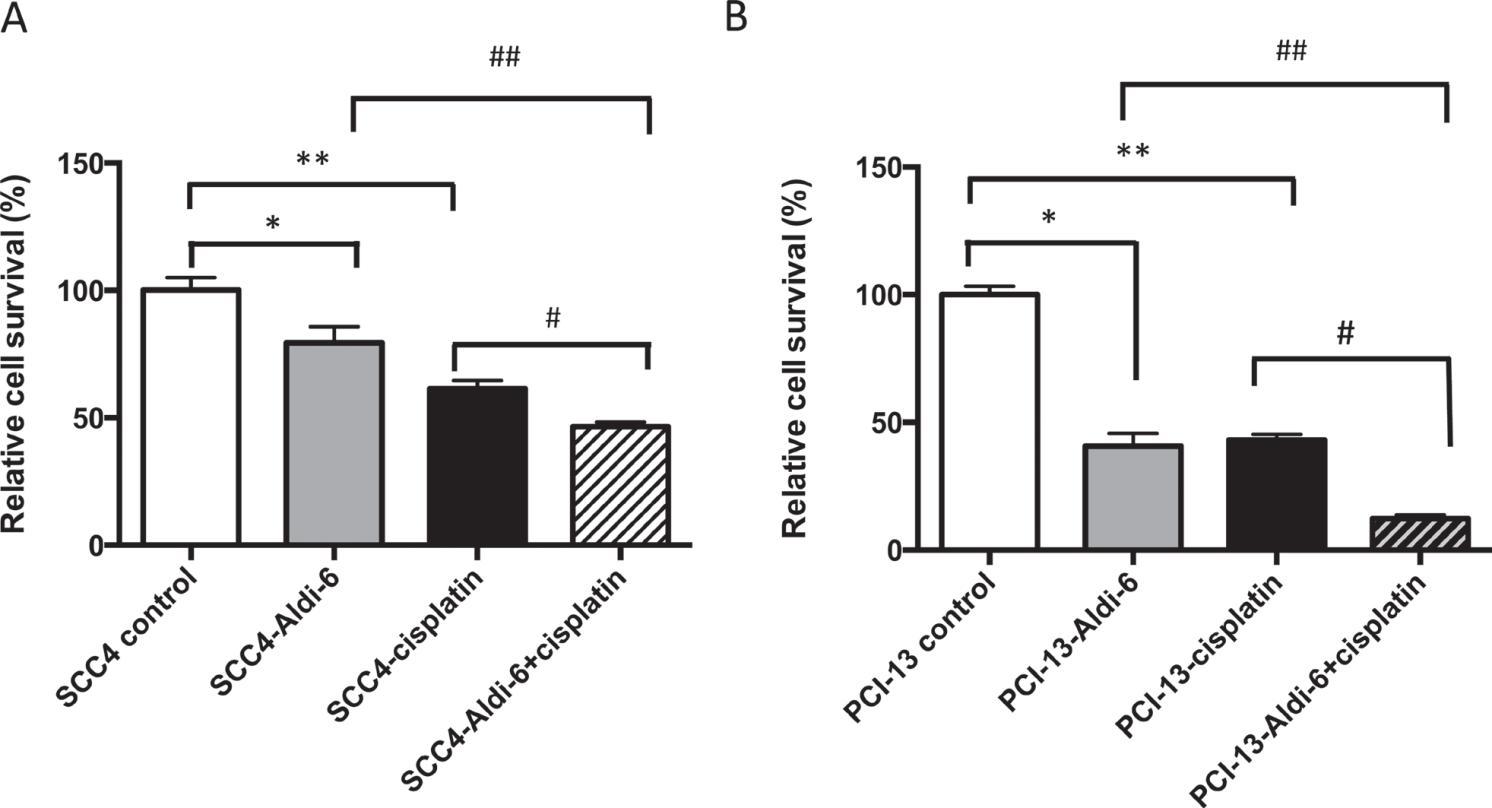
Aldi-6 decreases cell viability of HNSCC cells (**A**) SCC4 and (**B**) PCI-13 cells were treated with Aldi-6 and/or cisplatin for two consecutive days. Then, the cells were analyzed on the fourth day. Cell viability was quantified using MTT assay. Results represent mean ± SEMs of 8–16 replicates. *p < 0.05 and **p < 0.0001 *vs*. control; ^#^*p* < 0.005 *vs*. cisplatin only group; ^##^ *p* < 0.05 *vs*. Aldi-6 only group (*t*-test).

Since Aldi-6 was found to have profound ALDH inhibitory activity (Figure 5), we assessed the ability of this compound to sensitize HNSCC cells to cisplatin. We treated SCC4 and PCI-13 cells with a combination of Aldi-6 (30 μM) and/or cisplatin (15 μM) and quantified cell viability by MTT assay (Figure 7). In SCC4 cells, we observed a 21% reduction in cell viability with Aldi-6 alone and a 40% reduction in cell viability with cisplatin alone. The combination of Aldi-6 and cisplatin treatment resulted in an even greater reduction in cell viability (Figure 7A). This reduction in viability parallels an increase in ROS levels (Supplementary Figure 6). Conversely, we show that N-acetylcysteine, an antioxidant, can rescue the impact of Aldi-6 and/or cisplatin on cell survival, indicating that the combined impact of Aldi-6 and cisplatin is through an increased level of ROS (Supplementary Figure 7). A similar reduction in viability was observed in PCI-13 cells as well (Figure 7B). Thus, the combination of Aldi-6 and cisplatin has a profound effect on the cell viability of HNSCC.

### Aldi-6 reduces tumor growth rate *in vivo*

**Figure 8:**
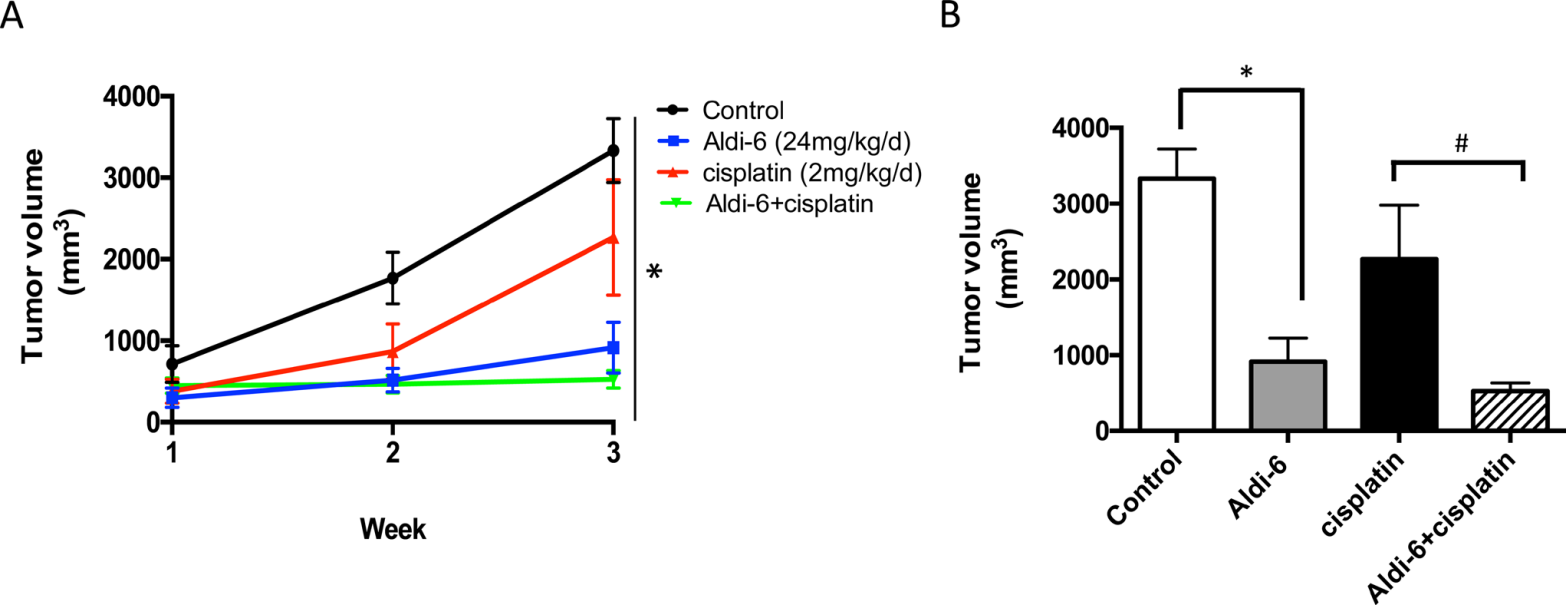
Aldi-6 reduces HNSCC tumor growth rate *in vivo* (**A**) SCC4 cells (2 × 10^6^) were subcutaneously injected into the flanks of NOD-*scid* IL2Rgamma^null^ mice (*n* = 3–6 per group). Mice were treated systemically with Aldi-6, using implantable osmotic mini pumps (24 mg/kg/day) for continuous delivery of the compound. Cisplatin was administered by weekly i.p. injection (2 mg/kg) for 3 weeks. Tumor size was measured weekly for three weeks. One-way ANOVA analysis was performed on the final tumor size (*p < 0.05). (**B**) Quantification of the final tumor volumes (*p < 0.05 *vs*. control; and ^#^*p* < 0.05 *vs*. cisplatin, *t*-test, *n* = 3–6 per cohort).

To assess the anti-tumor therapeutic potential of Aldi-6, SCC4 cells were subcutaneously injected into the flanks of immunodeficient NOD-*scid* IL2Rgamma^null^ (NSG) mice. The mice were treated systemically with Aldi-6, using implantable osmotic mini pumps (24 mg/kg/day) for continuous delivery of the compound. Cisplatin was administered by weekly i.p. injection (2 mg/kg) for 3 weeks, and tumor size was monitored. We observed that Aldi-6, administered as a single agent, reduced tumor growth more effectively compared to the control or cisplatin treated cohorts (Figure 8A). Aldi-6 alone reduced the final tumor volume compared to control by 60% (Figure 8B). Treatment with both Aldi-6 and cisplatin reduced the final tumor volume by 75% compared to the tumors treated with cisplatin alone. Aldi-6 appeared to have more significant effects *in vivo*, compared to what was observed *in vitro* (Figure 7), indicating that additional anti-tumor mechanisms may be involved. Importantly, no systemic toxicity was observed during the treatment with Aldi-6. Specifically, no mortality or body weight loss was observed during the study (Supplementary Figure 8).

## DISCUSSION

ALDH isozymes are responsible for oxidizing intracellular reactive aldehydes and protecting cells from ROS-induced oxidative insult [11, 12, 15, 30], but the role of ALDH in the cisplatin chemosensitivity in HNSCC cells has not been investigated in detail. We hypothesized that inhibition of ALDH activity can effectively increase the oxidative insult from cisplatin and potentiate the efficacy of chemotherapy.

Our data indicate that ALDH3A1 plays a role in cisplatin-resistant cell survival in HNSCC and that inhibition of this enzyme may be a useful strategy in the cisplatin refractory context. The expression of ALDH1 alone or with the expression of cell surface markers CD44 or CD133, has been used to enrich a cell population with chemoresistant and stem-cell like properties in head and neck squamous cancer cells [20, 32], lung [22], colon cancer [33] and in breast cancer [24, 34, 35]. Here, we demonstrate that ALDH3A1 is upregulated in human primary HNSCC tumors and in HNSCC cell lines following exposure to cisplatin, suggesting a functional role for this isozyme in cisplatin resistance.

In this study, we investigated a novel small molecule ALDH inhibitor, Aldi-6, that we identified by a high throughput screen. Based on the common core structure, Aldi-6 may inhibit ALDH3A1 by forming a covalent adduct with the active site cysteine (243) residue in ALDH3A1, similar to Aldis 1-3 [30]. The exact molecular mechanism of inhibition will be investigated in the future studies. Aldi-6 could inhibit ALDH3A1 induction by cisplatin in HNSCC, and there was a corresponding reduction in cellular survival. This indicates that ALDH3A1 expression is an important part of the survival mechanism of HNSCC exposed to cisplatin. Similarly, it was recently observed that gastric epithelial cancer cells with high ALDH activity were shown to be resistant to cisplatin or 5-Fluorouracil [36]. We observed an enhanced reduction in cell viability with combination treatment of Aldi-6 and cisplatin (Figure 7). Further, Aldi-6 alone had profound effects on cell viability *in vitro* and tumor growth inhibition *in vivo*, indicating that Aldi-6 alone may be an effective agent against HNSCC. Recently, CB29 was identified as another ALDH3A1 specific inhibitor (but not for ALDH1A1 or 2, up to 250 μM). However, CB29 inhibited ALDH3A1 with an IC_50_ of 16 μM, which is 16 times greater than that of Aldi-6.

One of molecular mechanisms underlying the cytotoxic activity of cisplatin is through increased levels of intracellular ROS; these arise from the generation of highly reactive mono- and di-aquated form of cisplatin, which interact with and deplete endogenous nucleophilic antioxidants such as reduced glutathione, methionine and metallothioneins [8–10, 37]. The cancer stem cell populations in human and mouse breast tumors have been reported to have lower ROS and higher scavenging capacity compared to the non-cancer stem cell population, and this greater ability to handle oxidative stress resulted in a greater resistance to DNA damaging irradiation [38]. Recently, ovarian clear cell carcinoma was reported to be enriched in the ALDH-high population in cells with a higher scavenging capacity for reactive oxygen species [39]. Thus, it can be envisioned that ALDH inhibition through compounds like Aldi-6 may be a useful strategy to address chemo- and radiation-resistant malignant cancer cells possessing high ALDH expression.

Our *in vivo* experiment demonstrates that a short-term Aldi-6 infusion results in reduction in tumor growth with better efficacy than cisplatin treatment alone; however, because the tumors reached a size limit in a relatively short period, we could not study the effect of long-term treatment with these agents. During the treatment period, the impact of Aldi-6 alone was greater than that seen *in vitro*. It is well known that the level of ROS is increased in solid tumors, because when a solid tumor grows, the center becomes distant from the blood vessels leading to the metabolic stress, and the appearance of hypoxic and glucose depleted areas are common features [40]. Thus, even in the absence of cisplatin, there is likely to be increased mitochondrial production of ROS due to hypoxia. It is possible that in the *in vivo* setting, Aldi-6 may have more profound effects if the tumor is already under high oxidative stress. This possibility is actually extremely intriguing and provides rationale for future investigation of Aldi-6's single agent activity against squamous cell carcinoma. Of note, Aldi-6 was very well tolerated with no mortality or body weight loss observed during the treatment, and this provides further rationale for exploring this agent in preclinical models of HNSCC.

## MATERIALS AND METHODS

### Human cell lines and tumor specimens

The human HNSCC cell lines used in this study (SCC4 and PCI-13) have been previously described [41, 42]. The SCC4 cell line was obtained from ATCC. The PCI-13 cell line was a kind gift from Dr. Jennifer Grandis (Dept. of Otolaryngology, University of California, San Francisco). Cells were cultured at 37°C under a humidified 5% CO_2_ and 95% air atmosphere in DMEM/F12 containing 10% fetal bovine serum (FBS) with 1% penicillin/streptomycin (10,000 U/ml penicillin and 10 mg/ml streptomycin). Human tongue tumor tissues were obtained from patients who underwent surgical resection of their tumors. All patients signed an informed consent approved by the Stanford Institutional Review Board.

### Antibodies and reagents

Aldefluor was purchased from Stem Cell Technologies (Vancouver, Canada), antibodies for ALDH1A1, ALDH2 and ALDH3A1 were purchased from Santa Cruz Biotechnology (ALDH1A1; sc-374076, ALDH2; sc-48837, ALDH3A1; sc-67309 (Santa Cruz, CA)) and GAPDH and β-tubulin antibodies were from Advanced immunology (MAb 6C5, Oakland Gardens, NY) and Cell Signaling (Beverly, MA), respectively. Cisplatin was from Enzo Life Sciences (ALX-400-040-M250, AnnArbor, MI). MTT (3-(4,5-dimethylthiazol-2-yl)-2,5-diphenyltetrazolium bromide) reagent was purchased from Millipore (CT01-5, Billerica, Massachusetts) and 2′,7′-dichlorodihydrofluorescin diacetate for ROS assay was from Sigma and Cell Bioabs (D6883, St. Louis, MO, and STA-342, San Diego, CA). Aldi-6 (3-(Dimethylamino)-4′-bromopropiophenone, an ALDH1A1, 2 and 3A1 inhibitor) and Alda-89 (S9652, 4-Allyl-1,2-methylenedioxybenzene, an ALDH3A1-activator, Sigma, St. Louis, MO) were identified by our high throughput screening of small molecules.

### Aldefluor ALDH activity analysis and flow cytometry

ALDH activity was determined using the Aldefluor assay per the manufacturer's instructions (Stem Cell Technologies, Vancouver, Canada). Cells (1 × 10^6^) were treated with either DMSO or Aldi-6 (30 μM for 48–72 hours) and resuspended in Aldefluor assay buffer containing the ALDH substrate, bodipy-aminoacetaldehyde (BAAA; 5 μM), for 45 minutes at 37°C. As a negative control for each treatment condition, cells were incubated with 15 μM diethylaminobenzaldehyde (DEAB), an ALDH inhibitor. Fluorescence activated cell sorting (FACS) was performed using a BD Aria II. Aldefluor fluorescence was excited at 488 nm and emission was detected using a standard fluorescein isothiocyanate (FITC) 530/40 nm band-pass filter. The ALDH^+^ population was determined relative to the corresponding DEAB treated control. Data were acquired using BD FACSDiva and analyzed with FlowJo version × 10.0.7.

### Isozyme specific ALDH activity assay

Recombinant ALDH isozymes were homogenized in the buffer as described below. Cofactor and substrate (NAD^+^ and acetaldehyde) were added in the reaction buffer and the increase in the level of NADH was observed over time by spectrophotometer. For a 2 ml assay, 1 ml of 100 mM NaPPi (final concentration at 50 mM NaPPi buffer (pH 9.0 (MW 446)), 0.5 ml of 10 mM NAD^+^ (2.5 mM NAD^+^), 20 μl of 1 M acetaldehyde (10 mM acetaldehyde), 20 μl of ALDH enzyme (10–100 μg protein) and 460 μl H_2_O were added and mixed. Absorbance (O.D.) was measured at A340 nm for 3–5 minutes (6.22 O.D. = 1 mmole of NADH measured with 1 cm width cuvette). We used samples with no acetaldehyde as a blank control. The homogenization buffer consisted of 1 ml of 1 M Tris HCl pH 8.0 (final concentration of 0.1 M Tris HCl), 0.1 ml of 1 M DTT (10 mM DTT (MW 154)), 2.3 ml of 87% glycerol (20% glycerol) and 6.5 ml of H_2_O with 0.1 ml of TrionX-100 (1%).

### ALDH in-gel activity assay

Isozyme specific activity of ALDH was also measured using isoelectric focusing (IEF) methods, as previously described [31]. Briefly, IEF is a method for separating protein on the basis of the isoelectric points. A particular protein will focus at a unique isoelectric point. Because ALDH1A1, 2 and 3A1 have different isoelectric points of 5.2, 4.9 and 6.0, respectively, the bands appear in different positions in a pH gradient corresponding to each isozymes’ isoelectric point, representing individual activity. The method for IEF contains three steps: a prefocusing step where the pH gradient is formed, a tissue sample application step and a focusing step. Tumor tissues were lysed in non-denaturing buffer (Tris HCl pH 8, 0.1M, 10 mM DTT, 20% glycerol and 0.5% TritonX 100). Homogenates were centrifuged at 100,000 g for 30 min. Equal amounts of protein samples were loaded (100 μg/4 μl) onto a pre-cast gel, and the gel was run immediately using PhastSystem equipment (GE, Upsala, Sweden). After focusing, the gel was stained with a 20 ml volume of staining solution containing substrate (0.2–0.5ml of 0.1 M acetaldehyde) and 2 ml of 10 mM NAD (NAD becomes NADH, which reacts with dye to turn blue), 2ml of 1 M Tris HCl, 1 ml of 10mM Nitroblue tetrazolium (NBT), 1 ml of phenazine methosulfate (PMS) 20 mM (dissolved in MeOH), and 13.5 ml H_2_O, for 30min in the dark for development.

### Western blot analysis

Cell lysates were prepared in RIPA/Lamelli buffer containing a Protease and Phosphatase Inhibitors Cocktail (Pierce Biotechnology Inc., Waltham, MA) and centrifuged at 5,000 g for 10 minutes at 4°C. Protein concentration was measured using a Bio-Rad Protein Assay (Bio-Rad Laboratories, Hercules, CA). Equal amounts of proteins (10–20 μg) from each sample were subjected to SDS/PAGE and transferred onto PVDF membranes (Invitrogen, Carlsbad, CA). Membranes were first probed with primary antibodies and subsequently with HRP-conjugated secondary antibodies. Protein bands were detected by a commercial SuperSignal West Pico Chemiluminescent HRP Substrate detection reagent (Pierce Biotechnology Inc., Waltham, MA) or a chemiluminescent reagent containing luminol.

### Knockdown of ALDH3A1 in SCC4 cells

Cells were seeded in 6-well plates 15 to 18 hours before the start of the treatment. Then, cells were transduced with a combination of four different shRNA lentiviral vectors to knockdown ALDH3A1 (ABM, Inc., Richmond, Canada; iLenti for ALDH3A1). Transduced cells were selected by puromycin and were used for Western Blot and MTT assays.

### Colorimetric MTT assay for cell proliferation

MTT assay reagents from Millipore were used for cell proliferation. The assay was carried out according to the manufacturer's instructions. Cells were seeded at 5,000 cells per well in a 96-well plate 15 to 18 hours before the start of the treatment. Cells were treated with the compounds on days 1 and 2 and then 0.01 ml of MTT (Millipore CT01-5, 50 mg/ml in PBS) solution was added on day 4 to each well, and the cells were incubated for 4 hours at 37°C in the dark for the cleavage of MTT to occur. Color development solution (isopropanol with 0.04 N HCl, 0.1 ml each) was then added and mixed thoroughly. Within an hour, absorbance was measured at 570 nm and at a reference wavelength at 630 nm. Data are calculated as absorbances measured at 570 nm subtracted by those measured at 630 nm and were reported in arbitrary units and expressed as percent of control.

### *In vivo* tumor growth assay

Six-week-old male NSG mice were from a breeding colony from Dr. Michael Clarke's lab (Stanford University). All mice were kept under standard temperature, humidity, and timed lighting conditions and were provided with mouse chow and water ad libitum. All animal experimentation protocols were approved by the Stanford University Animal Care and Use Committee. Two million SCC4 cells were injected subcutaneously in the flank in a mixture of 1:1 PBS and Matrigel (Becton Dickinson). Aldi-6 and cisplatin treatment began when the tumors reached a group average of 200–300 mm^3^ after 1–2 weeks. Aldi-6 was delivered by osmotic pumps (Alzet model 2004, 0.25 ul/hr, 28 days, at 24 mg/kg/day) implanted in the mouse's flank with drugs lasting for 3 weeks to deliver Aldi-6 or vehicle control. Cisplatin was injected intraperitoneally and was given once a week at 2 mg/kg/day for 3 weeks dissolved in saline. Tumor volume (mm^3^) was calculated using the equation 0.52 × (width (mm))^2^ × length (mm).

### Statistics

Data are expressed as mean ± SEM. Statistical analysis of *t*-tests and one way ANOVA were used to compare the different number of samples analyzed by Westernblot, flow cytometry, MTT, tumor measurements and various ALDH isoform expressions in cells and from human tissues. A value *p* < 0.05 is considered to be significant.

### Reactive oxygen species (ROS) assay

Cells were seeded at 5,000 cells per well in a 96-well plate 15 to 18 hours before the start of the treatment. Aldi-6 or Alda-89 was prepared in DMSO/PBS mix and cisplatin was prepared fresh by adding 1 mg of cisplatin into 1 ml of saline. TritonX-100 Cells were treated two times with the compounds on days 1 and 2 and then on day 4, were washed with PBS. Cells were incubated with 2′,7′-dichlorodihydrofluorescein diacetate (DCFH-DA, Oxi-select kit, STA-342, Cell Biolabs, San Diego, CA) (20 μM-1 mM) at 37°C in the dark 30 min to 1 hr. Cells were then washed again with PBS and lysed. 2′,7′-dichlorodihydrofluorescein (DCF) fluorescence was measured within 30 minutes using a BioTek FL-600 plate reader (BioTek Instruments, Winooski, Vt., USA) at 485 nm excitation and 530 nm emission wavelengths. Data were expressed in nM of DCF as calculated from the standard curves.

## SUPPLEMENTARY FIGURES


