# Supplementary material for: Targeting aldehyde dehydrogenase activity in head and neck squamous cell carcinoma with a novel small molecule inhibitor
Source: Oncotarget. 2017 Apr 10;8(32):52345–56. doi: 10.18632/oncotarget.17017 (PMC5581033; doi:10.18632/oncotarget.17017)
Supplement: Supplementary file 1 [file oncotarget-08-52345-s001.pdf]

Targeting aldehyde dehydrogenase activity in head and neck squamous cell carcinoma with a novel small molecule inhibitor

Supplementary Materials

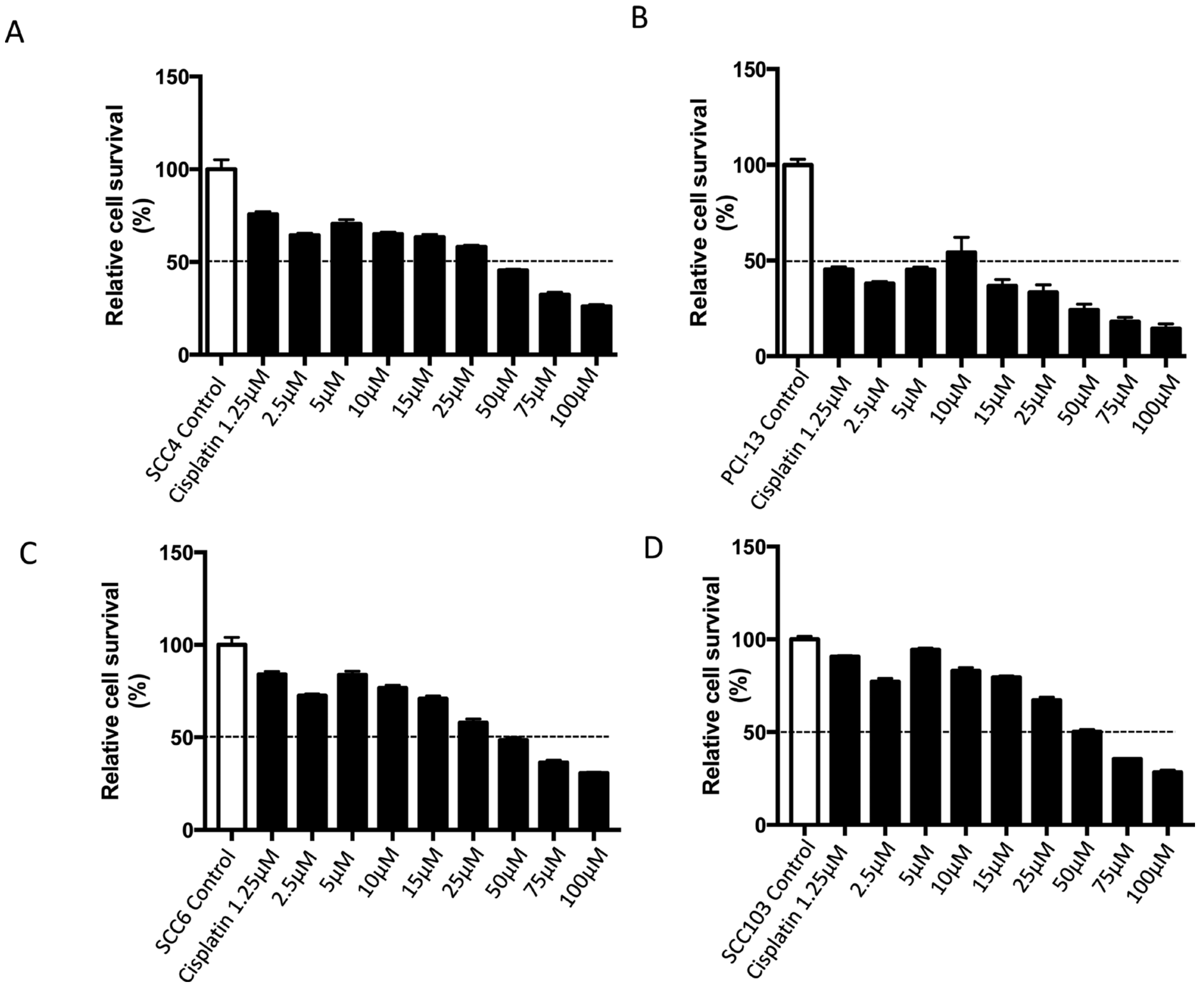

Supplementary Figure 1: Cell survival in cisplatin-treated HNSCC cells. (**A**) SCC4, (**B**) PCI-13, (**C**) SCC6 and (**D**) SCC-103 cells were seeded at 5,000 cells per well in 96 well plate and were treated with cisplatin (0–100 µM). Cell viability on the fourth day was measured by MTT assay. Results represent mean ± SEMs of 8–24 replicates.

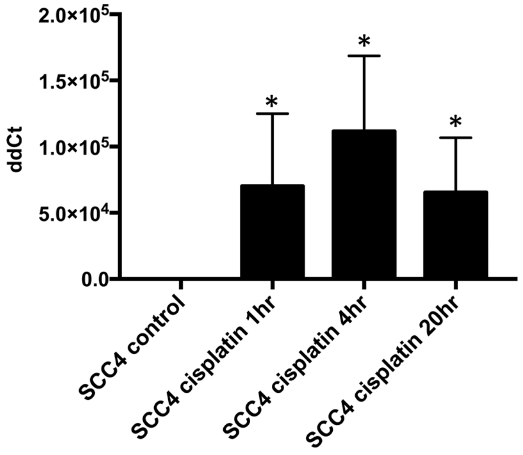

Supplementary Figure 2: Transcription of ALDH3A1 is induced as early after cisplatin treatment in SCC4 cells. SCC4 cells were treated with cisplatin (15 μM) for 1–20 hours. Then, the RNA was extracted and gene expression of ALDH3A1 was measured using qPCR assay. The level of gene expression is shown in ddCt compared to that of the control cells (**p* < 0.05 *vs*. untreated control).

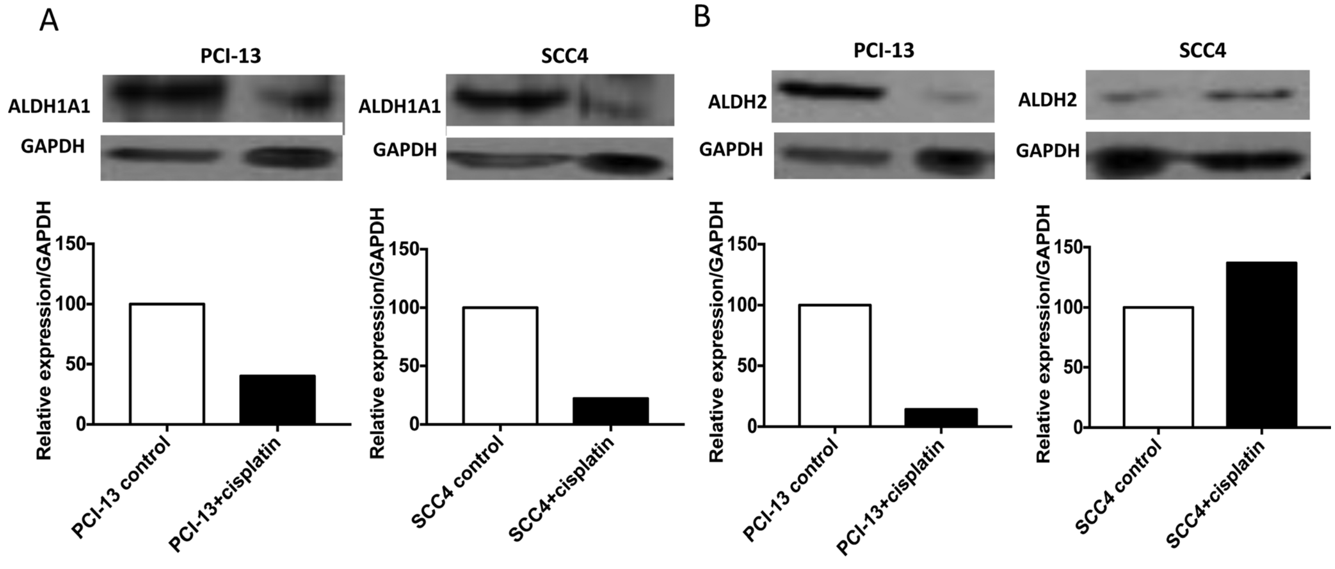

Supplementary Figure 3: ALDH1A1 and 2 levels in cisplatin-treated HNSCC cells. Protein levels of (**A**) ALDH1A1 and (**B**) ALDH2 were measured by Western blot and quantified by densitometry after a two-day treatment of cisplatin (15 μM) in PCI-13 and SCC4 cells.

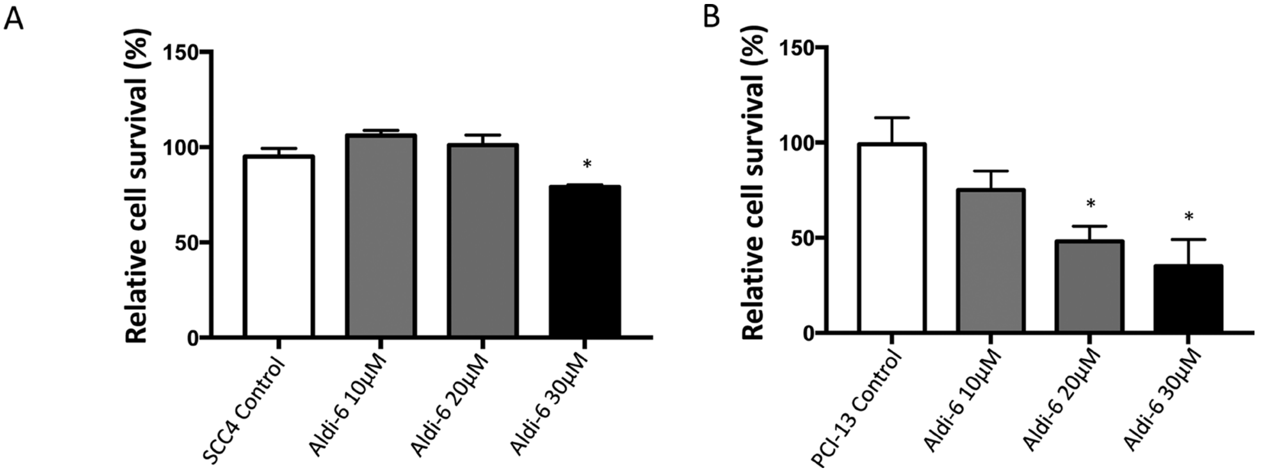

Supplementary Figure 4: Cell survival after Aldi-6 treatment in SCC4 and PCI-13 cells. Cells were treated with different concentrations of Aldi-6 for two consecutive days. Then, the cells were analyzed on the fourth day. Cell viability was quantified using MTT assay. Results were expressed in relative cell survival as percent of control (*$p < 0.05$ vs. untreated control, *t*-test).

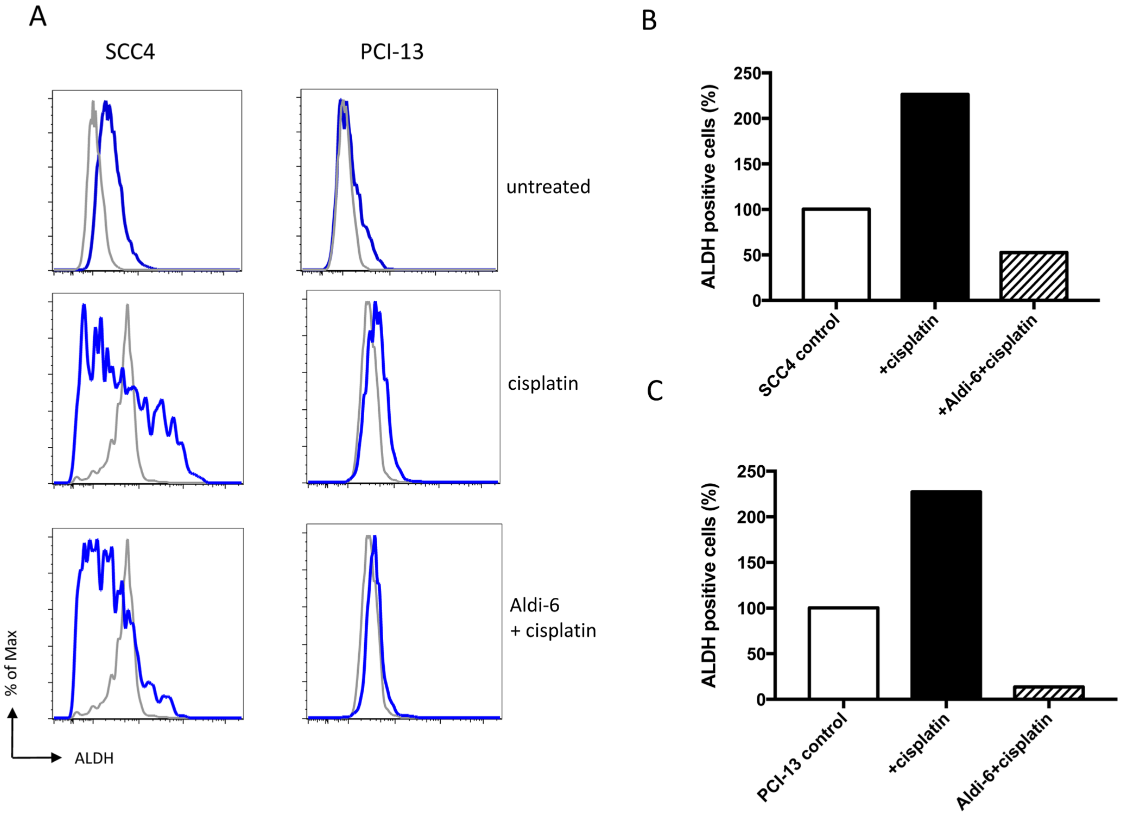

Supplementary Figure 5: Aldi-6 inhibits ALDH activity induced by cisplatin (15 µM) in HNSCC. (**A**) Representative FACS analyses of ALDH activity (measured by Aldefluor assay) of SCC4 and PCI-13 cells. Cells were seeded at 4 million cells in 10 cm plates 15 to 18 hours before the start of the treatment. After a two-day treatment of cisplatin (15 µM) and/or Aldi-6 (30 µM), ALDH activity was measured in the surviving cells on the fourth day by Aldefluor assay. Grey line represents the DEAB-treated negative control for each treatment condition. Blue line represents the ALDH activity of each sample. (**B**–**C**) Changes in the ALDH positive cells were quantified and expressed as percent of control.

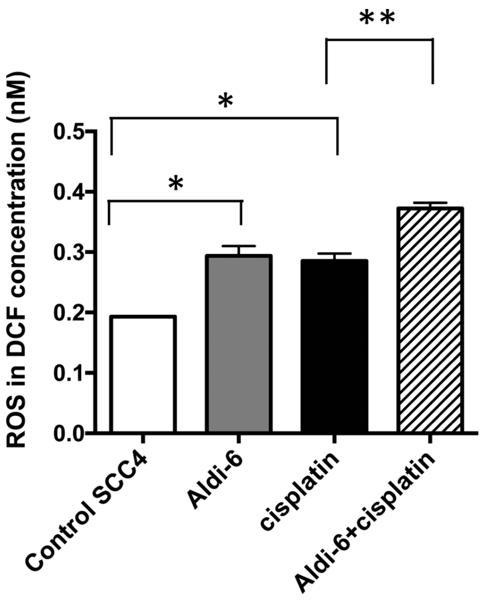

Supplementary Figure 6: Aldi-6 increases ROS production in cisplatin-treated cells. SCC4 cells were seeded at 5,000 cells per well in 96 well plates and were treated with Aldi-6 and cisplatin for two days. Then, the cells were analyzed on the fourth day (see Methods). We observed an increase in measured ROS levels in SCC4 cells exposed to cisplatin alone and to Aldi-6 alone. The combination of Aldi-6 and cisplatin increased ROS levels even further compared to the cells treated with cisplatin alone (*$p < 0.05$ vs. control; and **$p < 0.05$ *vs*. cisplatin, *t*-test, $n = 3$–6 per cohort).

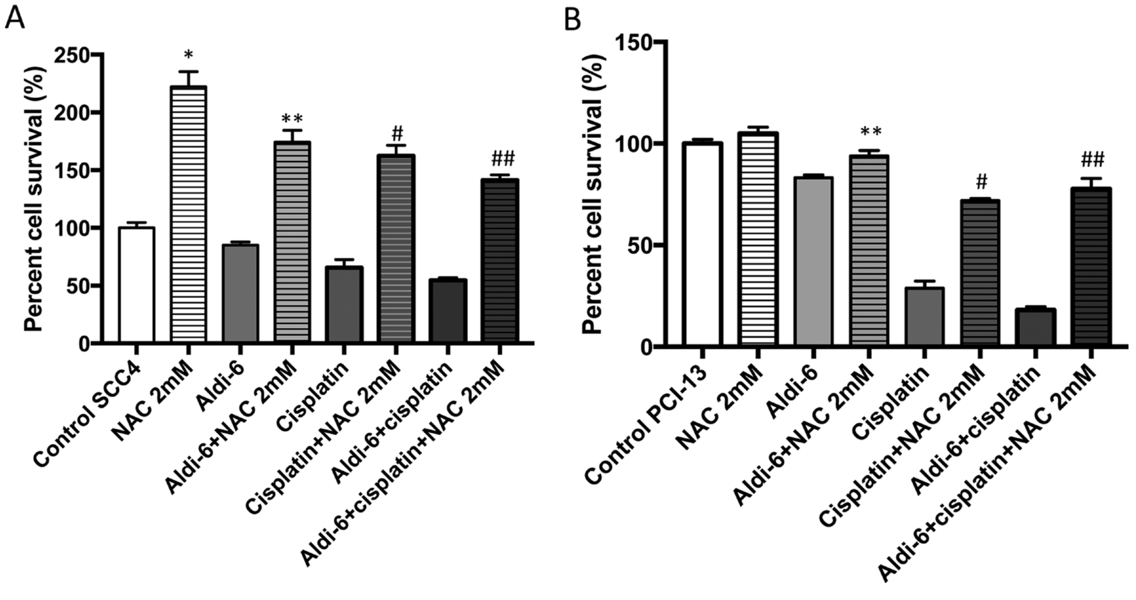

Supplementary Figure 7: N-acetylcysteine rescues cell viability of Aldi-6 or cisplatin treated HNSCC cells. (**A**) SCC4 and (**B**) PCI-13 cells were treated with N-acetylcysteine (2 mM) and Aldi-6 and/or cisplatin for two consecutive days. Then, the cells were analyzed on the fourth day. Cell viability was quantified using MTT assay. Results represent mean ± SEMs of 8 replicates (*, **, $^{\#}$, $^{\#\#}p < 0.05$ *vs*. respective control without NAC (*t*-test)).

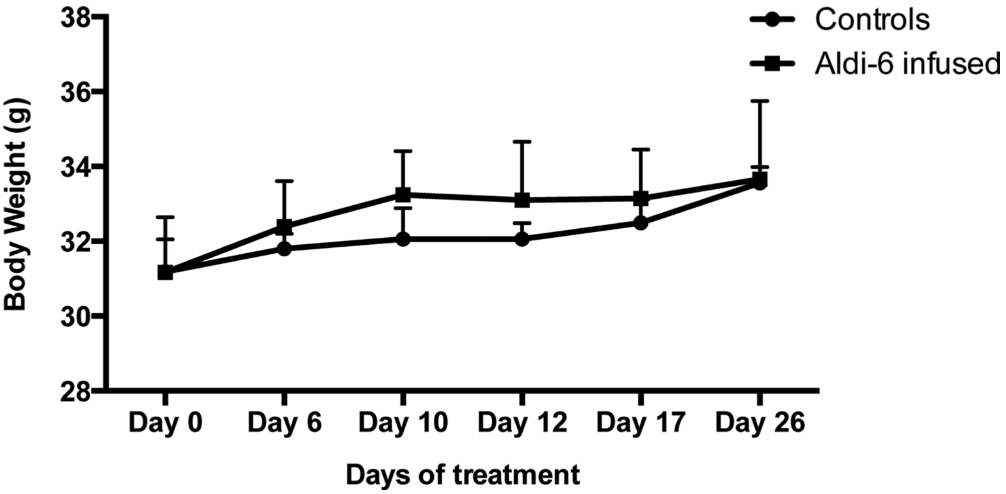

Supplementary Figure 8: Body weight changes in the Aldi-6 treated mice. C57BL/6 mice were treated systemically with vehicle control or Aldi-6 (*n* = 3 each) for 3 weeks, using implantable osmotic mini pumps (24 mg/kg/day) for continuous delivery of the compound. The results show no significant body weight change in the Aldi-6 treated mice compared to the controls.
